# Supplementary material for: Health System Response during the European Refugee Crisis: Policy and Practice Analysis in Four Italian Regions
Source: Int J Environ Res Public Health. 2020 Jul 29;17(15):5458. doi: 10.3390/ijerph17155458 (PMC7432017; doi:10.3390/ijerph17155458)
Supplement: Supplementary file 1 [file ijerph-17-05458-s001.zip › untitled folder/Table S1.pdf]

Table S1 - Checklist used for semi-structured interview

**Items**	**Notes**
Area	City/Local health organization
Health workers involved for the data collection	Role in the healthcare services
Iter → care pathway	Describing reception system, actors, and roles
First medical examination (ME)	When, Who, Where, Why, What
Screening	When, Who, Where, Why, What
Immunizations' program	When, Who, Where, Why, What
Health records	Individual health records and data transmission
Access to healthcare	Fees, entitlement, other specific modality, or admission to the system
Continuity of care after the first ME	When, Who, Where, Why, What
Challenges, critical issues, suggestions	Open
