# Supplementary material for: Health System Response during the European Refugee Crisis: Policy and Practice Analysis in Four Italian Regions
Source: Int J Environ Res Public Health. 2020 Jul 29;17(15):5458. doi: 10.3390/ijerph17155458 (PMC7432017; doi:10.3390/ijerph17155458)
Supplement: Supplementary file 1 [file ijerph-17-05458-s001.zip › untitled folder/Table S2.pdf]

Table S2. Summary of international and national recommendations according to the Mladovsky framework and selected items for policy analysis

Area/Recommendations	Items/target
**Data collection**<br>Identify immediate needs during episodes of mass international migration; Promote the inclusion of migrant variables in existing data collection systems [21]; Use informative system to collect data during all the phase of the reception) [35];<br><br>Improve the collection of and access to information on the health status of refugees, ASs and migrants, their modifiable risk behaviors and access to health care; Disaggregation and comparability of data is required; Enhance epidemiological surveillance capacities to include migrant-sensitive data; Use innovative approaches, including surveys and qualitative methods [21];<br><br>Promote the portability of health data in accordance with national law [21];<br><br>Produce progress reports on the health status of refugees, asylum seekers and migrants [21];	Continuative and computer migrant-sensitive collection system;<br><br>Typology of data;<br><br>Portability/Transmissibility;<br><br>Report/scope of collection;
**Population groups**<br>Emphasis should be placed on improving the health of the most vulnerable, including children, pregnant women, adolescents, the elderly, people with disabilities and victims of torture; The health needs of UASC require special attention; Issues relating to sexual and reproductive health, family planning, gender-based violence and rape Management, forced marriage and adolescent pregnancy, and mental health and care should be prioritized [21];<br>There is a need for the prevention and management of physical and psychological trauma and injury among refugees originating from countries affected by conflict and violence, as they are often exposed to the elements during their journeys; Some migrant women may wish to be cared for by female doctors, which could invoke issues of cultural sensitivity and gender-based equity [21, 35];	UASC;<br>Pregnant women;<br>Adolescents;<br>Elderly;<br>People with disabilities;<br>People with mental issue;<br>Victims of violence (any) and torture;
**Health issue addressed**<br><br>**CDs**<br>Screening for TB, malaria, STDs e parasitosis (Strongyloides e Schistosoma) is recommended during first ME, in the presence of symptoms or risk factors or origin from countries with a high prevalence [35];<br>Screening for HIV, HBV, HCV, LTBI is recommended during the phase of take in care, in the presence of symptoms or risk factors or origin from countries with a high prevalence [35];<br>Ensure, within available resources, appropriate immunization programs for refugees, ASs and migrants and close immunization gaps in recipient communities [21]; Vaccination, is recommended for child from 0 to 14 y when documentation is uncertain, according with national immunization programs [35];<br>Vaccination for polio, diphtheria, tetanus, pertussis, measles, mumps, rubella, chicken pox, HBV, is recommended in adults with unknown immunization history [35];<br>Include refugees, ASs and migrants in any outbreak control measures taken [21];<br><br>**NCDs**<br>Provide, within available resources, early access to essential primary care, essential dental health, preventive health and health promotion services, and diagnosis and treatment services, to allow for the prevention, detection, treatment and monitoring of NCDs [21];<br>Initial screening – not limited to CDs – can be an effective public health instrument, but should be nondiscriminatory and non-stigmatizing and carried out to the benefit of the individual and the public; it should also be linked to accessing treatment, care and support. It is unlikely to be necessary if health systems are strong and capable [21];<br>It should be provided on a voluntary basis, and with ethical attention to confidentiality.	Screening during first ME: TB, malaria, STDs, parasitosis;<br><br>Screening during take in care: HIV, HBV, HCV, LTBI;<br><br>Immunization programs child (0-14) and adults (polio, diphtheria, tetanus, pertussis, measles, mumps, rubella, chicken pox, HBV);<br><br>ASs in IPC;<br><br>Screening, early access to essential primary care, accessing treatment, care, and support;<br><br>Counselling and health education; Screening for diabetes, hypertension,

Confidentiality and medical ethics should be enforced, and pre- and post-screening counselling should be provided [21];<br>Screening for diabetes, hypertension, anemic, cervical cancer, is recommended during first medical examination, in the presence of symptoms or risk factors or origin from countries with a high prevalence [35];<br>First ME must include evaluation of nutritional state, functionality of cardiorespiratory system, visual and auditory acuity, and active research of skin signs of ectoparasites and violence and torture [35];<br><br>**Vulnerability**<br>Migration increases exposure to psychosocial disorders, reproductive health problems, neonatal mortality, drug abuse, nutrition disorders, harmful alcohol use and exposure to violence. Limited access to health promotion, disease prevention and care during the transit and early insertion phases of migration increases the burden of untreated and complicated NCDs conditions [21];<br>Early detection and timely multidisciplinary integrated take in charge are strictly recommended; LHOs must define a specific and multidisciplinary diagnostic-therapeutic path oriented to health protection and rehabilitation of the victims of torture; Certification of vulnerability must be accessible and LHOs/Region should identify and publish suitable facilities [36];<br><br>**Maternal and child healthcare (MCH)**<br>Access to screening programs that are in place for the host population (for example, screening during pregnancy, for neonatal diseases and for school entry) should, however, be explicitly promoted for migrant populations [21];<br>Screening for pregnancy is recommended during the first ME, to activate a differentiated reception path and find eventual sexual violence [35];<br>Maternal health care must be guaranteed for all pregnant women, according with the national law and with the support of linguistic and cultural mediators [35];	anemic, cervical cancer; Screening for visual and auditory acuity, dental health;<br><br>Screening for psychosocial disorders, drugs and alcohol abuse, nutrition disorders; Screening for violence and torture, specific and multidisciplinary diagnostic-therapeutic-rehabilitation path;<br><br>Screening for pregnancy, access to screening programs that are in place for the host population, screening during pregnancy for neonatal diseases, access to maternal and neonatal health care;
**Part of health system targeted**<br><br>**Equity and quality of care**<br>Access to responsive, people-centered health systems is essential to ensure available health care for all refugees, ASs, and migrants throughout the migration journey. This implies overcoming formal and informal barriers to healthcare, such as language, administrative hurdles, lack of information about health entitlements, and meeting the needs of all people, without discrimination, including on cultural or religious grounds [36];<br>Health systems should aim to offer culturally sensitive health care, overcoming barriers such as language, access to interpreters, administrative hurdles, and lack of support for patient fees or for information about health entitlements. Systems should ensure support to refugees and migrants in navigating through the system [21];<br>Ensure that necessary health and social services are delivered, within available resources, to refugees and migrants in a gender-sensitive, culturally and linguistically appropriate way without stigma, through advocacy and the provision of cultural mediators and by enforcing, when necessary, laws and regulations that prohibit discrimination [21,35];<br>Provide, within available resources, early access to essential primary care, essential dental health, preventive health and health promotion services, and diagnosis and treatment services, to allow for the prevention, detection, treatment and monitoring of NCDs; Promote health literacy [21];<br>Access to diagnosis of TB, treatment and continuity of care is recommended, especially for people moving in other places or countries; Enhancing the continuity and quality of care received by population groups in all settings, with a particular emphasis on pregnant women, children and the elderly [21];<br><br>**Monitoring and governance**<br>Generate evidence and address the health needs of refugees, ASs and migrants, and of host populations, providing surveillance and health protection and community information and recognizing the need for integrated interventions based on the different needs of migrants as a whole, and among migrant populations, according to age, gender, culture, education, demographic factors and nature of trauma [21];<br>Health assessment is a tool to identify vulnerable groups, and emphasis should be placed on	Culturally sensitive health services, access to interpreters, provision of cultural mediators;<br><br>Overcome administrative hurdles;<br><br>Support for patient fees;<br><br>Information about health entitlements and support in navigating through the system;<br><br>Ensure early access, continuity, and quality of care (primary care, preventive care, health promotion services, prevention, detection, treatment and monitoring of NCDs, CDs, vulnerabilities, MCH);<br><br>Health assessment to identify vulnerable groups and health

improving the health of the most vulnerable [21];<br>Strengthening reporting and accountability structures and mechanisms [21]; Implementation of multidisciplinary diagnostic-therapeutic-rehabilitative path, must be monitored by LHOs and an annual quali-quantitative report must be given at the MoH [36];<br>Strengthen health information systems for improved data collection on refugee and migrant health [21];<br>Establish government focal points for refugee and migrant health issues with the authority and capacity to support the implementation of a coordinated and multisectoral national health policy to integrate migrant health in public health policies and plans and to cooperate with neighboring countries (sub regional platforms and/or bodies), in accordance with national legislation, priorities and circumstances [21];	profile;<br><br>Reporting of implementation, accountability, and data collection;<br><br>Government focal points and cooperation;
**Training, guidance, and support**<br>Training on health equity and human rights-based approaches is a key element for health professionals and relevant non-health actors. Patient-sensitive health systems may benefit from fostering active and effective community participation and empowerment of refugees and migrants [21];<br>Training and continuous update of social and health workers, are the most important tools for promoting the health protection and for guaranteeing adequate quality of care for the victims of torture [36];<br>Provide the necessary guidance, training, and support tools to enable health systems and public health staff, services and planners to understand and implement appropriate migrant-sensitive interventions, including CDs, NCDs, vulnerabilities interventions, prevention, management and screening [21,36];<br>Include, if feasible, qualified migrant health workers in the design, implementation and evaluation of migrant-sensitive health services and educational programs [21];<br>Include migration health in the graduate, postgraduate and continuous professional training of all health personnel, including support and managerial staff, with an emphasis on cultural mediation [21];	Community information;<br><br>Training and continuous update with health equity and human rights-based approaches, and specific focus (es. victim of torture);<br><br>Community participation;<br><br>Guidance, training, and support tools to migrant sensitive interventions on CDs, CDs, vulnerabilities;<br><br>Skilled health professional on migrant health/continuous professional training;<br><br>Migrants health workers;

Note: AS = Asylum seeker; CDs = Communicable diseases; HIV = Human Immunodeficiency Virus; HBV = Hepatitis B virus; HCV = Hepatitis C virus; IPC = Infection prevention and control; LHO = Local health organization; LTBI = Latent tuberculosis infection; ME = Medical examinations; MCH = Maternal and child health ; MoH = Ministry of Health; NCDs = Non-communicable diseases; STDs = Sexual transmitted diseases; TB = Tuberculosis; UASC = Unaccompanied and separated children; Y = Years;
