# Supplementary material for: Health System Response during the European Refugee Crisis: Policy and Practice Analysis in Four Italian Regions
Source: Int J Environ Res Public Health. 2020 Jul 29;17(15):5458. doi: 10.3390/ijerph17155458 (PMC7432017; doi:10.3390/ijerph17155458)
Supplement: Supplementary file 1 [file ijerph-17-05458-s001.zip › untitled folder/Table S3.pdf]

Table S3. Regional policies analyzed.

	Emilia-Romagna	Lazio	Toscana	Veneto
**Laws, Decree**	Law 5/2004 of 24th March 2004, norms for integration of immigrants [48];<br>Decree, Regularization of immigrant caregivers [49];<br>Decree, Elderly immigrants' enrolment in Regional Health Service [50];<br>Decree, Health protection of children of undocumented migrants [51];<br>Decree, Access to primary health care for children of undocumented migrants [52];<br>Decree, Healthcare for minors and their caregivers [53];<br>Decree, Healthcare coverage exemption for minors coming from Ukraine, Belarus, and Saharawi [54];<br>Decree, Agreement between RHS and medical syndicate for the access to primary healthcare for minors and their caregivers [55];	Law 10/2008 of 25th May 2008, norms for the promotion and the protection of immigrant' rights [62];<br>Decree, transposition of Government-Regions Agreement on immigrant's healthcare [63];<br>Decree, enrolment in Regional Health Service of refugees and asylum seekers [64];<br>Decree, mandatory enrolment in Regional Health Service of refugees (including humanitarian protection) asylum seekers [65];<br>Decree, health care for asylum seekers [66];<br>Decree, enrolment in Regional Health Service of refugees and asylum seekers [67];<br>Decree, enrolment in Regional health services of refugees and asylum seekers coming from other regions [68];<br>Decree, annual enrolment in Regional Health Service of refugees and asylum seekers [69];<br>Decree, monitoring the implementation of the transposition of Government-Regions Agreement on immigrants' health care [70];	Law 40/2005 of 24th February 2005, norms for Regional Health Service [78];<br>Law 41/2005 of 24th February 2005, Integrated system of interventions and services for the protection of social citizenship rights [79];<br>Law 29/ 2009 of 9th June 2009, norms for the accommodation, the integration, and the protection of foreign citizens [80];<br>Decree, transposition of Government-Regions Agreement on immigrants' health care [81];<br>Decree, extra-UE elderly immigrants' enrolment in Regional Health Service [82];<br>Law 84/2015 of 28th December 2015, norms for RHS, modifications to law 40/2005 [83];<br>Decree, n. 1304 of 27th November 2017. Norms concerning international protection and regional immigration plan [84];<br>Decree, DGRT n.1000 of the 10th September of 2018. Protocols for migrant health care [85];	Law 55/1982 of 15th December of 1982, norms for social care [93];<br>Law 9/1990 of 30th January 1990, norms concerning immigration [94];<br>Law 1/2012 of 17th April 2012, statute of Veneto [95];<br>Decree, Regional observatory of immigration [96];
**Plans, protocols**	Protocols, healthcare coverage exemption [56];<br>Indications for Local Health Organizations for migrant health care [57];<br>Indications to Local Health Organizations for migrant health care national program [58];<br>2017-2019 Regional Social and Health plan [59];<br>Indications to Local Health Organizations for migrant	2008-2010 Regional Health Plan [71];<br>2010-2012 Regional Health Plan [72];<br>Guidelines, access to care for extra-UE immigrants [73];<br>Indications, health care of migrants arrived by sea and hosted in regional facilities [74];<br>Indications, health care of migrants arrived by	Guidelines, access to care for foreign citizens [86];<br>Guidelines, access to care for foreign citizens. Notes [87];<br>Protocols, healthcare coverage exemption for unaccompanied minors [88];<br>Protocols, healthcare coverage exemption [89];<br>Protocols, healthcare coverage exemption for	Guidelines, access to care for foreign citizens [97];<br>2012-2016 Regional Social and Health plan [98;]<br>2014-2018 Regional plan for preventive care [99];<br>Protocols for infectious disease prevention and control related to the large influx of immigrants [100];

	healthcare national program [60];<br>Indications to Local Health Organizations for migrant healthcare. Immunization program and LTBI screening on accommodation facilities [61];	sea in transit through the region [75];<br>Indications of regional hub for healthcare of displaced migrants [76];<br>Indications of healthcare for asylum seekers [77];	unaccompanied minors [90];<br>Protocols, healthcare coverage exemption for asylum seekers [91];<br>2018-2020 Regional Social and Health plan [92];	
