# Supplementary material for: Health System Response during the European Refugee Crisis: Policy and Practice Analysis in Four Italian Regions
Source: Int J Environ Res Public Health. 2020 Jul 29;17(15):5458. doi: 10.3390/ijerph17155458 (PMC7432017; doi:10.3390/ijerph17155458)
Supplement: Supplementary file 1 [file ijerph-17-05458-s001.zip › untitled folder/Table S4.pdf]

Table S4 - Policy analysis: data collection

	Emilia-Romagna	Lazio	Toscana	Veneto
**Continuative and computer migrant-sensitive collection system**	Provision of regional computer system for health facilities involved in migrant and AS health care [58,60];<br><br>Provision of data collection of first ME and health care through checklist on regional computer system [57-58,60];<br><br>Collection of ASs' data about access to health care through specific new variables and inclusion in ordinary data system [57-58,60];	Use of a defined checklist/ protocol for ME [74-77];	Computerization of the first ME [85];<br><br>Use of a specific checklist for the ME [85];	Not present;
**Typology of data**	Provision and monitoring of data of first ME and health care for ASs [58,60];<br><br>Identification of each AS by a personal code [57-58,60];<br><br>Inclusion of ASs in epidemiological surveillance by LHOs [57-58,60];	Data collection and monitoring of first ME and health care of ASs [74-77];<br><br>Data disaggregated [74-77];<br><br>Inclusion of ASs by LHOs in epidemiological surveillance [74-77];	Data collection and monitoring of first ME and health care of ASs [85];<br><br>Inclusion of ASs in epidemiological surveillance and regional health report [85, 92];	Collection of data regarding vaccinations performed, type and batch of vaccine and potential adverse events, both for children and adults [93-95];<br><br>Updating of the documentation certifying the vaccination status with a personal vaccination certificate [93-95];
**Portability/ transmissibility**	Personal health records follow ASs movement [57-58,60];	Not present;	Not present;	Not present;
**Report/aim of collection**	Production by RHA of a periodic report of activities and transmission of information to all the stakeholders involved in ASs' assistance [48,57-58,60];	Not present;	Collection and production of data for syndromic surveillance [85];<br><br>Observation, monitoring, analysis to recognize health status and specific health needs [79, 80, 86];	Not present;

Note: ASs = Asylum seekers; LHOs = Local health organizations; ME = Medical examination; RHA = Regional health authorities;
