# Supplementary material for: Health System Response during the European Refugee Crisis: Policy and Practice Analysis in Four Italian Regions
Source: Int J Environ Res Public Health. 2020 Jul 29;17(15):5458. doi: 10.3390/ijerph17155458 (PMC7432017; doi:10.3390/ijerph17155458)
Supplement: Supplementary file 1 [file ijerph-17-05458-s001.zip › untitled folder/Table S5.pdf]

Table S5. Policy analysis: population groups

	Emilia-Romagna	Lazio	Toscana	Veneto
**UASC and adolescents**	Entitlement to the NHS for all the children at arrival in the region [48,51-55,57-58,60];	Entitlement to the NHS for all the children at arrival in the region [62-69];	Entitlement to the NHS for all the children at arrival in the region.<br>Rights to entitlement to NHS also for irregular migrants' sons [78-81, 86-88, 90];	Entitlement to the NHS for all the children at arrival in the region [93-95];
**Maternity**	Protection of pregnancy and maternity with the equal rights of Italian citizens [48,51-55,57-60];	Protection of pregnancy and maternity with the equal rights of Italian citizens [62-69];	Protection of pregnancy and maternity with the equal rights of Italian citizens [78-81, 86, 87];	Protection of pregnancy and maternity with the equal rights of Italian citizens [93-95];
**Elderly**	Entitlement to the NHS for elderly immigrants [50];	Not present;	Entitlement to the NHS for elderly immigrants [82];	Not present;
**People with disabilities**	Not present;	Not present;	Not present;	Not present;
**People with mental issue**	Health protection for people with mental issue [59];	Not present;	Not present;	Not present;
**Victims of violence (any) and torture**	Health protection for victims of violence [59];	Not present;	Not present;	Not present;

Note: NHS = National Health Services; UASC = Unaccompanied and separated children;
