# Supplementary material for: Health System Response during the European Refugee Crisis: Policy and Practice Analysis in Four Italian Regions
Source: Int J Environ Res Public Health. 2020 Jul 29;17(15):5458. doi: 10.3390/ijerph17155458 (PMC7432017; doi:10.3390/ijerph17155458)
Supplement: Supplementary file 1 [file ijerph-17-05458-s001.zip › untitled folder/Table S6.pdf]

Table S6. **Policy analysis: health issue addressed**

	Emilia-Romagna	Lazio	Toscana	Veneto
**Screening, treatment and follow up for CDs**	ME at arrival [57-58,60];<br><br>Screening and eventual treatment for TB, TSD parasitosis, pediculosis, scabies, during first acceptance; [57-58,60]<br>Screening and possible treatment for LTBI during second acceptance [57-58,60-61];<br><br>Access to essential and emergency care, diagnosis, treatment and follow up must be guaranteed [57-60];	ME at arrival [74-77];<br><br>Screening and possible treatment for TB, parasitosis, during first acceptance [74-77];<br><br>Access to essential and emergency care, diagnosis, treatment and follow up must be guaranteed [62-63,74-77];	ME at arrival [85];<br><br>Inclusion in general population screening when entitled [83, 86, 87];	ME at arrival [100];<br><br>Screening and possible treatment for TB, scabies, and Polio during first acceptance [100];<br>Screening and eventual treatment for LTBI during second acceptance [100];<br><br>Access to essential and emergency care, diagnosis, treatment and follow up must be guaranteed [93-95, 98-100];
**Immunization**	Child and adult vaccination in accordance with the applicable law within the framework of general vaccine campaign [57-61];	Child and adult vaccination in accordance with the applicable law within the framework of general vaccine campaign [62-63,71-72, 74-77];	Child and adult vaccination in accordance with the applicable law within the framework of general vaccine campaign [83, 86, 87];	Child and adult vaccination in accordance with the applicable law within the framework of general vaccine campaign [93-95, 98-100];
**Screening, treatment and follow up for NCDs**	First ME: complete clinical evaluation and active research of previous and novel conditions; Blood test and urine test if needed [57-58,60];<br><br>Guarantee of access to essential and emergency care, diagnosis, treatment and follow up [57-60];	First ME: complete clinical evaluation and active research of previous and novel conditions; Blood test and urine test if needed [74-77];<br><br>Guarantee of access to essential and emergency care, diagnosis, treatment and follow up [62-63,71-72,74-77];	First ME: complete clinical evaluation and active research of previous and novel conditions; Blood test and urine test if needed [85];<br><br>Guarantee of access to essential and emergency care, diagnosis, treatment and follow up [80, 81, 86, 87];	Not present
**Multidisciplinary diagnostic-therapeutic-rehabilitation path for vulnerabilities**	Provision by LHOs of access for prevention, treatment, and rehabilitation for drug abuse [58];	Not present;	Not present;	Not present;
**Maternal and child health (MCH)**	Protection of pregnancy and maternity with the equal rights of Italian citizens [48,57-60];<br><br>Conduction of Screening for pregnancy	Protection of pregnancy and maternity with the equal rights of Italian citizens [62-63,71-72, 74-77];	Protection of pregnancy and maternity with the equal rights of Italian citizens [80, 81, 86, 87];<br><br>Entitlement to the NHS for all the children at	Entitlement for minors to all scheduled vaccination [98-100];

	during first ME [57-58,60];<br>Identification of MCH units for migrants in each LHO [48];		arrival in the region [80, 81, 86, 87];	
**Counselling, health education, health promotion**	Not present;	Not present;	Not present;	Not present;

Note: CDs = Communicable diseases; LHO = Local health organization; LTBI = Latent tuberculosis infection; ME = Medical examination; MCH = Maternal and child health; NCDs= Non-communicable diseases; NHS = National health service; STD = Sexual transmitted diseases; TB = Tuberculosis;
