# Supplementary material for: Health System Response during the European Refugee Crisis: Policy and Practice Analysis in Four Italian Regions
Source: Int J Environ Res Public Health. 2020 Jul 29;17(15):5458. doi: 10.3390/ijerph17155458 (PMC7432017; doi:10.3390/ijerph17155458)
Supplement: Supplementary file 1 [file ijerph-17-05458-s001.zip › untitled folder/Table S7.pdf]

Table S7. Policy analysis: part of health system targeted

	Emilia-Romagna	Lazio	Toscana	Veneto
**Overcome barriers in access to care**	Presence of cultural mediation services by LHOs [48,57-58,60];<br><br>Exemption from charge after formal asylum request for 6 months, renewable until the entitlement to RHS [56-58,60];<br><br>Guarantee of access to regional health insurance after formal asylum request [56-58,60];	Presence of cultural mediation services by LHOs [62-69,74-77];<br><br>Exemption from charge after formal asylum request for 6 months, renewable until the entitlement to RHS [62-69,73-77];<br><br>Guarantee of access to regional health insurance after formal asylum request [62-69,73-77];	Presence of cultural mediation services by LHOs [78-80, 83];<br><br>Exemption from charge for 12 months for ASs, as unoccupied;<br>Before the regularization, exemption from charge, following the national law [87-91];<br><br>Health entitlements and support in navigating through the system [78-80, 83];<br><br>Information measures to facilitate access to healthcare towards irregular migrants to improve knowledge and access [78-80, 83];	Presence of cultural mediation services by LHOs [100];
**Comprehensive primary health care and health promotion**	Guarantee by LHOs of health protection and early access to care; Right to access for ASs to RHS as well as host community [51-58,60];<br><br>Definition of dedicated services of LHOs to provide essential services to migrants and ASs [48];	Guarantee by LHOs of health protection and early access to care; Right to access for ASs to RHS as well as host community [62-69,73-77];<br><br>Definition of dedicated services of LHOs to provide essential services to migrants and ASs [76];	Guarantee by LHOs of health protection and early access to care; Right to access for ASs to RHS as well as host community [80-84, 89, 90];	Guarantee by LHOs of health protection and early access to care; Right to access for ASs to RHS as well as host community [93-95,97];<br><br>Definition of dedicated services of LHOs to provide essential services to migrants and ASs [93-95];
**Monitoring and governance**	RHA must produce a periodic report of activities and information should be transmitted to all the stakeholders involved in the ASs assistance [48,58];<br><br>Annual Report of health status and access to care of migrant and AS is provided by epidemiological regional center [48];	RHA make up a regional government point in collaboration with the NGOs local forum [62-63];	Production of regional report on migrants' health status to recognize migrants' health needs [80-87];<br><br>Regional service (Global health center - CSG) as coordination structure for health cooperation and migrants' health [83, 84];<br><br>Coordination with LHOs but also with	Not present;<br><br>Annual Report of health status and access to care [96];<br><br>Local Authorities predisposes and coordinate local integration plans and coordination taskforce<br>[93-95];

	LHOs must collect specific accountability for healthcare provided to AS [58];<br><br>Provision of healthcare and assistance in coordination and cooperation with other institutional stakeholders and NGOs to guarantee health protection and address Social determinants of health [48];		epidemiological regional center (ARS), other regional institutions, associations, volunteering aiming at sharing information, projects, actions and to produce an annual report [79, 80, 83, 84, 92];	
**Continuous training, guidance, and support to implement migrant sensitive interventions**	HCWs must be skilled on medicine of migration and migration and health [57-58,60];<br><br>Guidance, training, and support tools must be provided by LHOs to all HCWs [56];	Skilled HCWs on migrant health/continuous professional training [62-63, 74-77];<br><br>Guidance, training, and support tools must be provided by LHOs to all HCW [62-63,73];	Provision of specific education and skills for HCWs and development of cultural competencies within health services [79, 80, 83, 84, 92];	Technical group for "initiatives in migrant health care" must provide training and technical support to HCWs of LHO [93-95];<br><br>Guidance and support for acceptance center hygienic standards [100];

Note: ASs = Asylum seekers; HCW = Health workers; LHO = Local health organizations; NGO = Non-governmental organizations; RHA = Regional health authorities; RHS = Regional Health service;
